# The Influences of Soluble Phosphorus on Hydration Process and Mechanical Properties of Hemihydrate Gypsum under Deep Retarding Condition

**DOI:** 10.3390/ma15072680

**Published:** 2022-04-05

**Authors:** Puyue Fan, Mingtao Zhang, Min Zhao, Jiahui Peng, Kai Gao, Jing Huang, Wei Yi, Cong Zhu

**Affiliations:** 1 College of Materials Science and Engineering, Chongqing University, Chongqing 400045, China; 202009131200@cqu.edu.cn (P.F.); zmt@cqu.edu.cn (M.Z.); 201909021019@cqu.edu.cn (K.G.); 202109021112@cqu.edu.cn (J.H.); 20113639@cqu.edu.cn (W.Y.); 2 School of Civil and Architectural Engineering, Yangtze Normal University, Chongqing 408100, China; 3 College of Civil Engineering, Chongqing University of Arts and Sciences, Chongqing 402160, China; congzhu@cqwu.edu.cn

**Keywords:** hemihydrate gypsum, soluble phosphorus, hydration process, mechanical properties, mechanism

## Abstract

Phospho-gypsum is an industrial solid waste discharged from the phosphate production process. The waste includes complex impurities such as phosphoric acid and its salts, fluoride, and organics. Usually, retarders are mixed in gypsum-based building materials to extend setting time. Although the effects of the impurities on hydration properties and the mechanical strength of calcined gypsum have been analyzed, the impact and mechanism of soluble phosphorus on the phospho-gypsum under retardation is yet to be defined. In this study, we employed thermogravimetry (TG), X-ray diffraction (XRD) and scanning electron microscopy (SEM) to evaluate the hydration kinetics, phase transformation, structure, and morphology of the calcined gypsum. The data showed that the retarder or soluble phosphorus prolonged the setting time. A single retarder considerably shortened the initial setting time from 95 min to 60 min, even at the lowest dosage of 0.1 wt.% soluble phosphorus. In addition, drying flexural and compressive strengths were markedly decreased. On the other hand, the induction period was advanced with extension of acceleration and deceleration stage. SEM results indicated that the crystal morphology of the gypsum changed from a long to short column or block. An EDS analysis showed that phosphates were concentrated on the surface of gypsum crystals.

## 1. Introduction

Phospho-gypsum is a by-product of phosphoric acid production. Approximately 3~5 tons of phosphor-gypsum are produced during the production of 1 ton of phosphoric acid [1]. The global cumulative emissions of phosphor-gypsum is approximately 6 billion tons with an increasing rate of 150 million tons/year. It is projected that the total amount of phosphor-gypsum will double by 2025–2045 [2,3]. Utilization of phosphor-gypsum is one of the most important issues in China for ecological security protection and resource management. Phospho-gypsum is mainly composed of CaSO_4_·2H_2_O, and other complex impurities such as soluble phosphorus, soluble fluorine, organics, or alkali metal salts. [4,5]. Impurities have various negative effects on the performance of phosphate building materials [6,7], such as fluctuation of setting time, low strength, and unstable quality, which compromises effective utilization phospho-gypsum in China. In fact, soluble phosphorus is one of the most harmful impurities in phospho-gypsum since it extends setting time and reduces the material strength which result in coarsening of dihydrate gypsum crystals [8,9]. Forms of soluble phosphorus in phospho-gypsum include H_3_PO_4_, H_2_PO_4_^−^, and HPO_4_^2−^. Previous data demonstrated that the degree of exerting the negative effects was as follows: H_3_PO_4_ > H_2_PO_4_^−^ > HPO_4_^2−^ [10,11]. On the other hand, soluble phosphorus, fluorine, and organics were distributed on the crystal surfaces, which increased with an increase in particle size of the dihydrate gypsum [12]. During the hydration process, SO_4_^2−^ can be replaced by HPO_4_^2−^ into gypsum crystals to form a solid solution, which further affects the gypsum performance [13]. In addition, the crystal morphology of CaSO_4_·2H_2_O is modified by the impurities in phospho-gypsum, and needle-like gypsum crystals have a higher compressive strength than short columnar and irregular plate-shaped crystals [14,15]. To obtain high strength, the concentration of soluble phosphorus should be controlled below 87 mmol/kg in the phospho-gypsum [16].

In addition, since the hydration of calcined gypsum is too fast, retarders such as alkaline phosphates, organic acids and proteins are commonly used for gypsum-based materials [17,18]. Previous studies have shown that alkaline phosphate exerts a high influence at a low dosage. In addition, there was a formation of insoluble calcium phosphate and deposition on the surfaces of hemihydrate particles because of a reaction between PO_4_^3−^ and Ca^2+^. The deposition prevents further dissolution of hemihydrate gypsum and eventually reduces system supersaturation [19,20,21]. Another study compared eight types of retarders and demonstrated that citric had the highest efficiency [22]. The crystal morphology of the dihydrate gypsum was transformed from column to block, which was associated with a sharp decline of strength [23]. Bone glue can be adsorbed on the surfaces of a newly formed crystal nucleus which further coarsens the crystal morphology [24]. Previous data showed that proteins have a good retarding effect on building plaster [25]. On the other hand, the faster the hydration speed of gypsum, the more the formation of needle-shape crystals, which leads to more overlaps between crystals and higher strength [26]. Longer setting time leads to severe loss of strength [27]. These data show that a protein is the best retarder, with highly effective and minimal strength reduction in gypsum.

The influence of impurities on hydration properties and mechanical strength of calcined gypsum have attracted considerable attention. However, data on the effects and mechanisms of soluble phosphorus on the hydration process and performance of phospho-gypsum under retardation remains scant. In this study, different amounts of soluble phosphorus were added into desulfurization calcined gypsum with the mixture of protein. We then investigated the effect of soluble phosphorus on the hydration process, mechanical properties, and mechanism of calcined gypsum. This study provides theoretical basis and data support for phosphor-gypsum utilization.

## 2. Materials and Methods

### 2.1. Raw Materials

**Figure 1:**
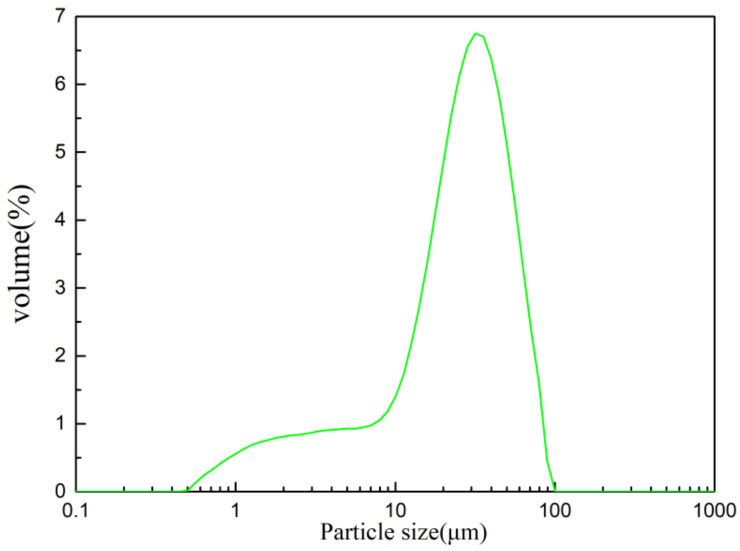
Particle size of the calcined gypsum.

Calcined gypsum is produced by Chongqing Yuju Environmental Protection Technology Co., LTD (Chongqing, China). The chemical composition and fundamental physical properties are as shown in Table 1 and Table 2, respectively. Analytical pure grade Na_2_HPO_4_ was from Chuandong Chemical (Group) Co. LTD (Chongqing, China), while the protein retarder was provided by Yingshan New Material Technology Co., LTD (Shanghai, China). We evaluated the mechanical properties according to GB/T 17669.3-1999 [28]. We also determined the particle size distribution of the calcined gypsum (d50 = 27.85 μm) (Figure 1) and its specific surface area, which was 385.00 kg/m^2^.

### 2.2. Methods

#### Sample Preparation

Gypsum specimens were prepared according to GB/T 28627-2012 [29]. Briefly, β- CaSO_4_·1/2H_2_O was slowly transferred into water in 30 s and then left for 30 s. The paste was quickly stirred for 60 s and then cast into standard steel molds to form specimens measuring 40 mm × 40 mm × 160 mm. After final setting, the specimens were demolded and stored in an indoor environment for 24 h. The specimens were further dried at 40 ± 4 °C to constant weight.

First, we determined the protein retarder dosage to an initial setting time of about 90 min for gypsum slurry at the standard consistency water consumption. On this basis, different amounts of Na_2_HPO_4_ were dissolved in water and while the protein retarder separately mixed with calcined gypsum. Thereafter, the mixed dry powder was poured into the Na_2_HPO_4_ solution. The amount of Na_2_HPO_4_ was selected based on the situation of the phospho-gypsum and the requirements of P_2_O_5_ (≤ 0.5%) as prescribed in the GB/T 23456-2018 [30]. We performed the experiments at seven different conditions as shown in Table 3. After hydration of the samples to the prescribed age, the paste was immersed in ethanol for 48 h to halt the hydration. The fragments after drying at 40 °C were used for the scanning electron microscope analysis and X-ray diffraction testing.

### 2.3. Measurements

#### 2.3.1. Determination of the Setting Time

Setting time was analyzed using the Vicat needle test according to GB/T 17669.4-1999 [31].

#### 2.3.2. Determination of the Mechanical Strength

Specimens were divided into two groups for natural strength at 1d or dry strength and tested according to GB/T 17669.3-1999 [28].

### 2.4. Characterization

To analyze the hydration process of hemihydrate with different concentrations of soluble P_2_O_5_, we measured the hydration heat through a TAM Air calorimeter from TA Instruments. To study the phase composition of a hardened gypsum sample, we analyzed the XRD patterns in an X-ray diffractometer (PANalytical X’Pert Powder, Panalytical B.V., Wuhan, China) using Cu Kα radiation at a scanning rate of 4°/min from 5° to 70°. The crystal morphology of the sample was analyzed by a SEM (Quattro S, Thermo Fisher, Shanghai, China) coupled with an energy dispersive X-ray spectroscopy (EDS). To further explain the hydration degree, we characterized the phase composition by simultaneous thermal analyzer (DSC/TG, TGA/DSC1/1600LF, Mettler Toledo, Shanghai, China).

## 3. Results

### 3.1. Influence of Soluble Phosphorus on Setting Time of the Calcined Gypsum

**Figure 2:**
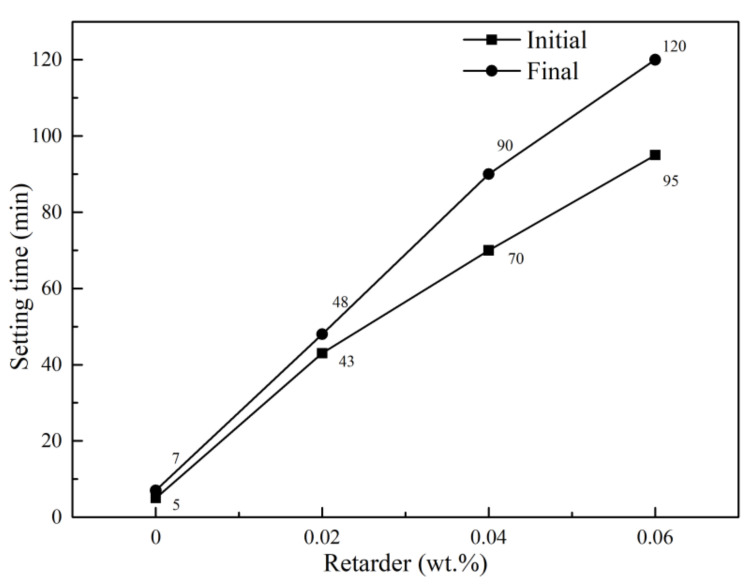
Effect of retarder on setting time of the calcined gypsum.

Setting time is one of the most important factors in building materials, which is related to the workability and the content of protein retarder. Different dosages of protein retarders were added into calcined gypsum and the influence of each group (S1–S4) was analyzed (Figure 2). The data showed that an increase in the retarder led to prolonged setting time. A dosage of 0.06 wt.% extended the initial setting time from 5 min to 95 min, and the final setting time was extended from 7 min to 120 min. The data also showed that the protein retarder had a more prominent effect on the initial setting time compared with the final setting time [24]. Protein retarders can dissolve in water and then form a colloid, which is adsorbed on the surfaces of CaSO_4_·2H_2_O crystals to form a protective layer preventing fast dissolution of hemihydrate gypsum. In addition, the surface energy was reduced by a colloid membrane leading to inhibition of the growth rate of CaSO_4_·2H_2_O crystal. In sync with previous data [32,33,34], the hydration process and the formation of the crystals network were slowed. In addition, the membrane became thicker and more stable with the increase in retarder, resulting in a longer setting time. The data showed that the protein retarder mainly prolonged the initial setting time (Figure 2).

**Figure 3:**
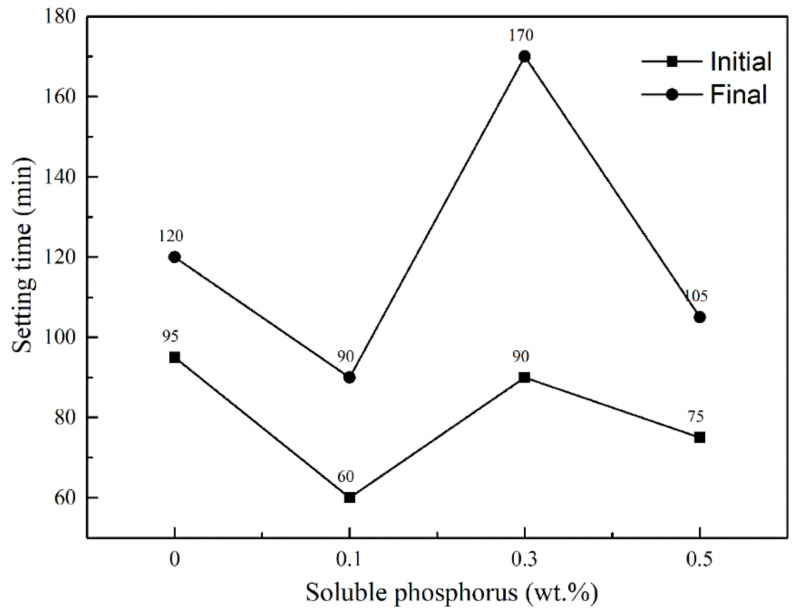
Influence of soluble phosphorus on setting time of the system.

To further simulate the industry practice, Na_2_HPO_4_ was added and mixed with 0.06 wt.% protein retarders. As shown in Figure 3, the initial setting time was shortened and the addition of Na_2_HPO_4_ extended the interval between initial and final setting time. At 0.3 wt.%, there was a decline in accelerating the effect with the increase in the content. When the content of soluble phosphorus was >0.3%, the accelerating effect was increased with the increase in the retarder dosage. Nevertheless, the setting time of calcined gypsum would be prolonged by a single soluble phosphorus or protein retarder [14]. Moreover, the retarding effect of the soluble phosphorus is much lower than the protein retarder, at the same concentration. However, the combination reaction between HPO_4_^2−^ and Ca^2+^ was stronger than the adsorption of colloid. On the other hand, the setting time of the system was influenced by the interaction of the two factors when the soluble phosphorus co-existed with the retarder. The change of setting time can be attributed to the fact that the addition of soluble phosphorus promoted HPO_4_^2−^ to replace SO_4_^2−^ in the dihydrate gypsum and influenced the setting time as well as strength by enriching soluble phosphorus on the surface of the dihydrate gypsum [35]. The adsorption and retarding effect of the protein retarder was greatly affected by HPO_4_^2−^, resulting in the change of setting time. Therefore, soluble phosphorus under deep retardation conferred opposite effects.

### 3.2. Influence of Soluble Phosphorus on Mechanical Strength of the Calcined Gypsum

**Figure 4:**
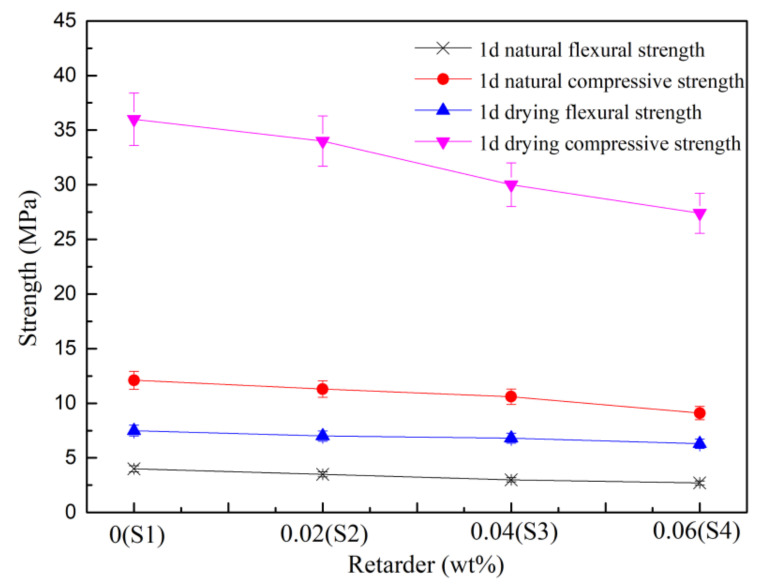
Influence of retarder on the strength of the calcined gypsum.

We defined the mechanical properties of gypsum hardened samples according to different dosages of the retarder (Figure 4). The flexural and compressive strengths of S1 (Calcined gypsum) were 4.0, 12.1 MPa and 7.5, 36.0 MPa for natural and absolute drying conditions, respectively. The retarder was harmful to mechanical strengths after the extension of the setting time, while the intensity substantially decreased with the increase in the retarder. When the initial setting time was extended to 95 min (S4), the flexural and compressive strengths of 1d natural and absolute drying condition were decreased to 2.7, 9.1 MPa and 6.6 MPa, 27.4 MPa, with the corresponding decline ratios of 32.5%, 24.8% and 12.0%, 23.9%, respectively.

**Figure 5:**
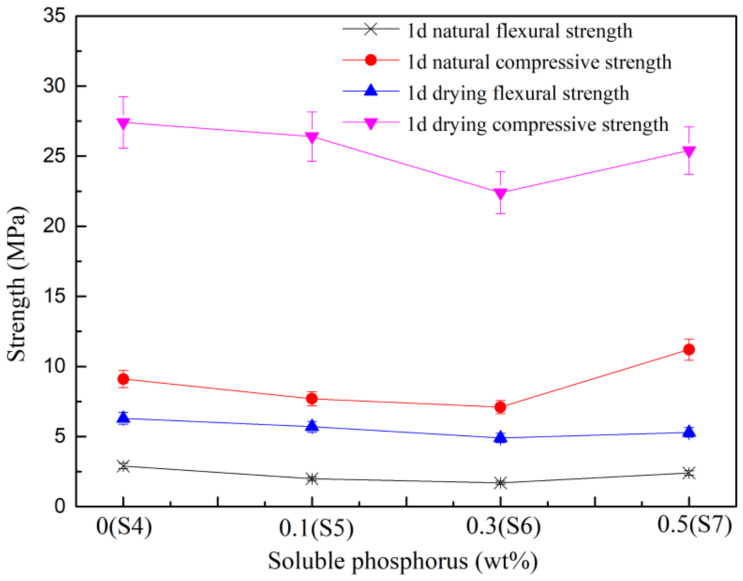
Influence of soluble phosphorus on strength of the system.

There was a significant change in the results after the addition of Na_2_HPO_4_ (Figure 5). When the dosage was lower than 0.3 wt.%, the strength continued to decline. Afterwards, the strength slightly increased at the dosage of 0.5 wt.% (S7) compared with S5 and S6, which was still lower than all samples from S1 to S4. These data demonstrated that sodium phosphate negatively affected the mechanical strength of phosphor-gypsum, independently of the retarder. The strength change was consistent with the setting time. The shorter the setting time, the higher the strength, which was associated with supersaturation of the liquid phase. In addition, shorter setting times were harder for the nucleation of gypsum crystals, which resulted in tiny gypsum crystals with more intersections and overlaps. On the contrary, previous data showed that there can be a longer dissolution-precipitation process and crystal growth process, leading to the formation of larger gypsum crystals [36,37].

### 3.3. Hydration Kinetics

**Figure 6:**
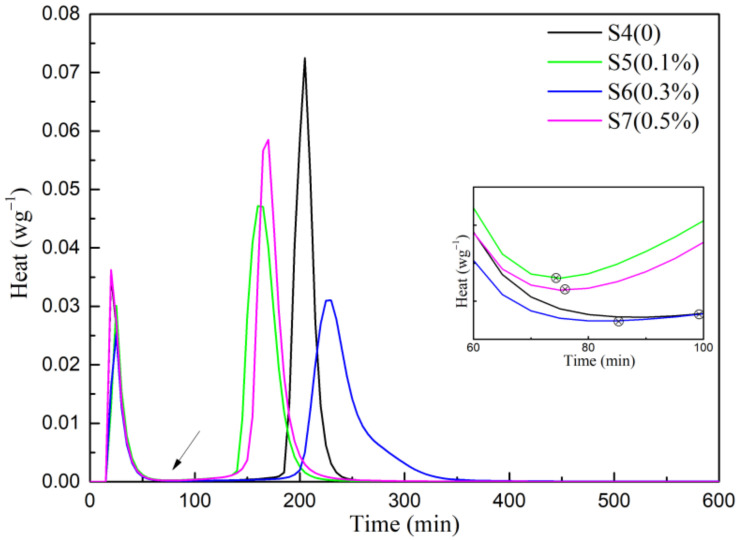
Rate of heat liberation-hydration time.

**Figure 7:**
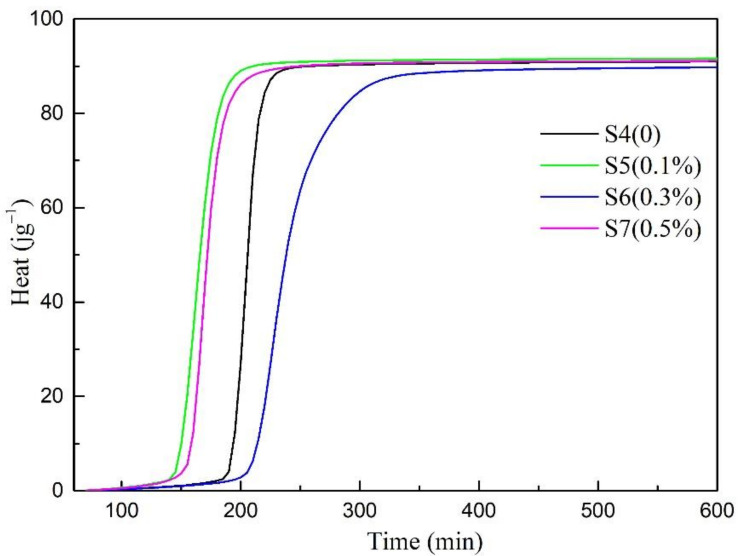
Hydration heat-hydration time.

To further define the mechanism of hydration of the β-CaSO_4_·1/2H_2_O with Na_2_HPO_4_, the rate of heat liberation and hydration heat appeared to be effective methods in the characterization of the different stages of β-CaSO_4_·1/2H_2_O hydration (Figure 6 and Figure 7).

According to the dissolution-nucleation-growth mechanism, the hydration process of β-CaSO_4_·1/2H_2_O can be divided into four stages: dissolution, induction, acceleration, and deceleration [38]. The data showed that there was immediate initial exothermic peak (peak I) due to initial dissolution of calcined gypsum powder, indicating the start of hydration. After peak I, the decreased rate of heat liberation was considered as an induction period during which gypsum nucleated, and ions of calcium and sulfate developed supersaturation [7]. The end of the induction period was advanced with the addition of Na_2_HPO_4_ as shown in Figure 6, which was equivalent to the initial setting time. This showed that soluble phosphorus accelerated the initial setting of the system. The induction period ended with the onset of the subsequent peak (Peak II). It is evident that hemihydrate gypsum containing Na_2_HPO_4_ had a longer peak II than S4 (0.06 wt.% retarder), because the protein retarder mainly prolonged the initial setting time. Once, the initial setting was achieved, the gypsum hydration process was accelerated, which can indicated the rapid achievement of final setting [24]. The hydration process was prolonged by the accumulation and precipitation of soluble phosphorus on the surface of CaSO_4_·2H_2_O crystal. As shown in Figure 7, the hydration heat in each group was roughly equal within 24 h, illustrating that the soluble phosphorus only changed the hydration kinetics without changing the hydration rate of the system.

### 3.4. DSC/TG Analysis

**Figure 8:**
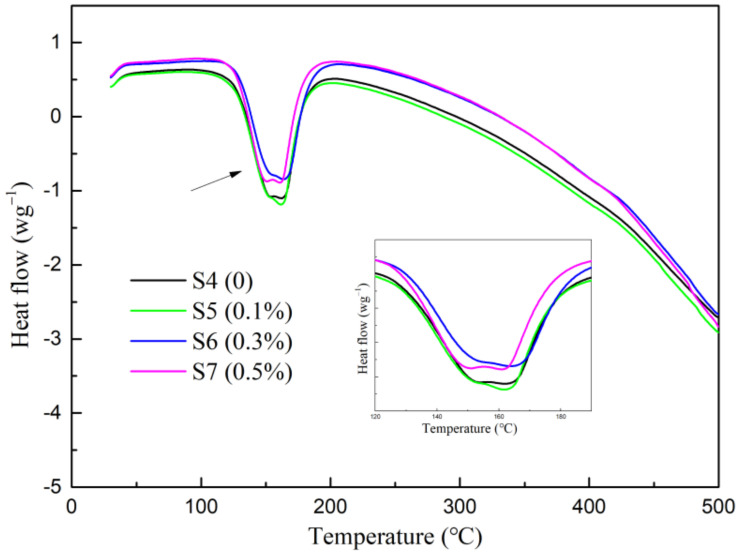
DSC curve.

**Figure 9:**
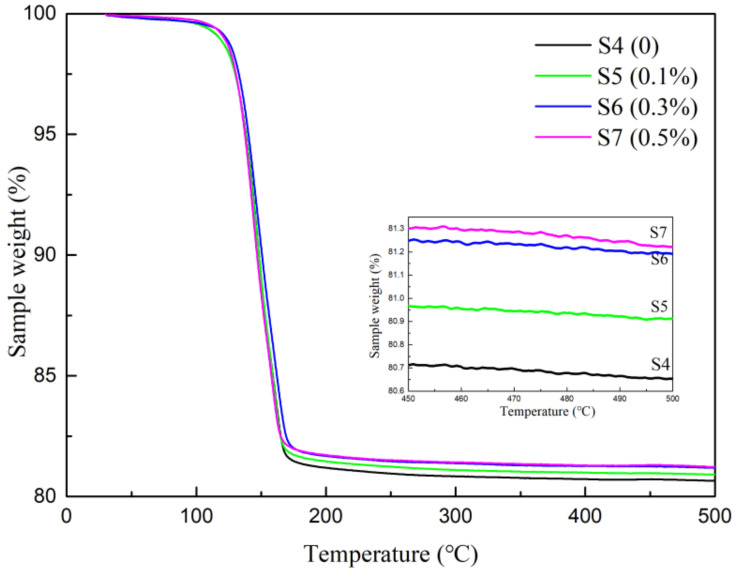
TG curve.

During the calcination process of the dihydrate gypsum, there was an occurrence of two dehydration reactions in a temperature range from 120 °C to 360 °C. The first step was the conversion of CaSO_4_·2H_2_O into CaSO_4_·1/2H_2_O, followed by dehydration of CaSO_4_·1/2H_2_O to CaSO_4_ [39]. The effect of soluble phosphorus on the dehydration process of the calcined gypsum was studied by DSC/TG as shown in Figure 8. The endothermic peaks were approximately 160 °C which meant that soluble phosphorus had little influence in the dehydration reactions. In addition, the weight losses from S4 to S7 were 19.35%, 19.09%, 18.81%, and 18.78%, respectively. The samples mixed with Na_2_HPO_4_ were decreased by degrees as shown by the TG curves (Figure 9). The production of calcium phosphate by the Ca^2+^ and HPO_4_^2−^ reaction led to the decrease in the amount of CaSO_4_·2H_2_O. Thus, the effect of a small amount of soluble phosphorus on the hydration rate of gypsum was insignificant.

### 3.5. XRD Analysis

**Figure 10:**
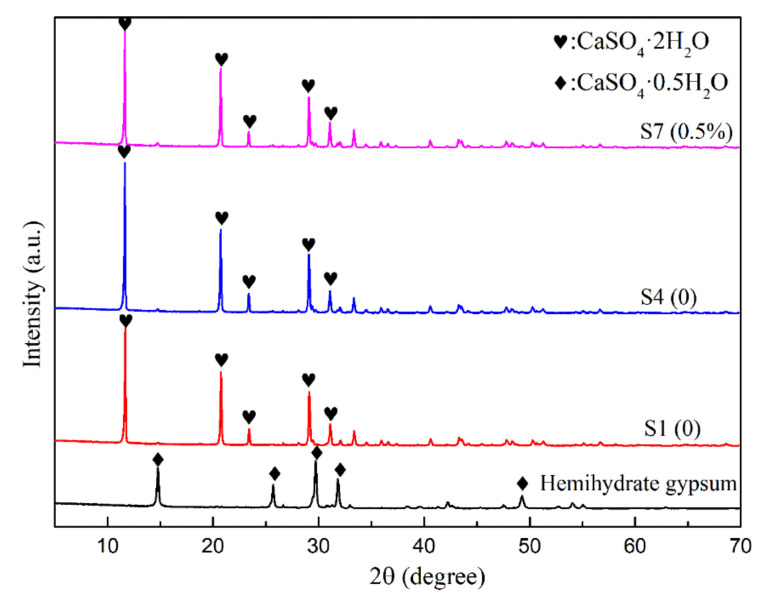
XRD pattens of the crystals.

The phase compositions were determined by XRD as shown in Figure 10. The main characteristic peaks of CaSO_4_·2H_2_O were identified at 2θ = 11.7°, 20.5°, 23.7°, 29.2°, and 31.2°. The location of diffraction angle and the peak intensity of the pure sample were largely consistent to Na_2_HPO_4_ samples. The data showed that HPO_4_^2−^ and SO_4_^2−^ can replace each other in the crystal structure of CaSO_4_·2H_2_O and can form a solid solution [40]. In other words, there is existence of calcium phosphate crystals of admixture samples. However, it was difficult to assess in the XRD spectra owing to the little content of Na_2_HPO_4_. Our results agreed with the DSC/TG findings.

### 3.6. SEM Crystal Structure

**Figure 11:**
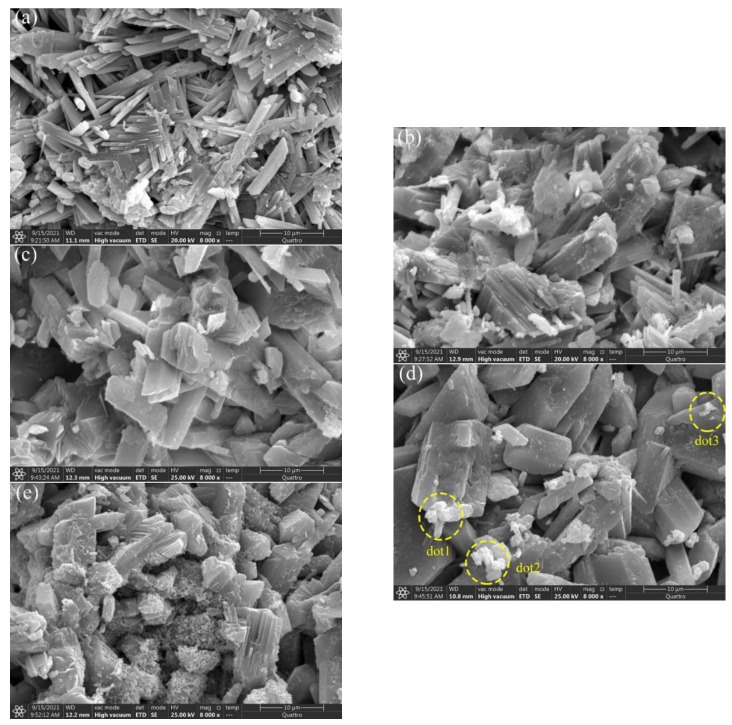
SEM images of the gypsum hardeners, (**a**) S1 (0% retarder + 0% P_2_O_5_); (**b**) S4(0.06wt.% retarder + 0% P_2_O_5_); (**c**) S5(0.06wt.%retarder + 0.1wt.% P_2_O_5_); (**d**) S6 (0.06wt.%retarder + 0.3wt.% P_2_O_5_); (**e**) S7(0.06wt.%retarder + 0.5wt.% P_2_O_5_).

The microstructure of the hardened sample was analyzed by SEM to assess the influence of the protein retarder and soluble phosphorus. The SEM data showed that the net-like and compact microstructure of the gypsum was composed of acicular and rod-like crystals of CaSO_4_·2H_2_O, with a length and diameter ratio of 8:1 as shown in Figure 11a. The crystals were closely overlapped and intersected, and the length of a single crystal was about 15 μm. Acicular CaSO_4_·2H_2_O crystals and related crystals which are capable of producing efficient cross junctions defined the high strength gypsum, especially high flexural strength gypsum [41]. The compressive strength of CaSO_4_·2H_2_O depended on the strength of the bond between the crystals [42]. The addition of the retarder transformed the crystal shape from long column and acicular to short column, while decreasing the length and diameter ratio, with reduction in overlap between crystals (Figure 11b). In addition, the retarder reduced the solution oversaturation and the crystal growth rate was delayed. The retarder adsorbed on the long axis of the gypsum leading to inhibition of the growth. As a result, the crystals were transformed into stumpy and coarse crystals, with more gaps between the crystals which resulted in a sharp decline of strength [43]. In addition, the addition of Na_2_HPO_4_ further coarsened the crystals with the increase in dosage as shown in Figure 11c–e. On the other hand, the shape was transformed into large stumpy and plate-like structures. The effective lap between the crystals was substantially reduced and the porosity was increased. At the 0.5 wt.% dosage, there was no existence of regular shape for the hydrate gypsum. Meanwhile, a film of small crystals was coated outside the crystal surface of CaSO_4_·2H_2_O, and minimum amount of P was detected by EDS analysis as shown in Table 4. These data inferred that the small crystals were calcium phosphate crystals. The microstructure was consistent with the decline of strength of the gypsum by both the retarder and Na_2_HPO_4_.

**Figure 12:**
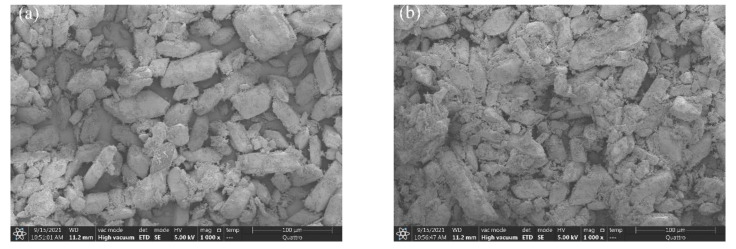
SEM analysis of gypsum hydrating for 10 min, (**a**) S4(0.06wt.%retarder + 0%P_2_O_5_); (**b**) S7(0.06wt.%retarder + 0.5wt.%P_2_O_5_).

The SEM analysis for 10 min of hydrated samples of S4 (retarder) and S7 (retarder and Na_2_HPO_4_) is shown in Figure 12. The data showed that the hydration rate of S4 sample was slower because of the retarder. Besides, there was less overlap between crystals at 10 min hydration than those of S7. Compared with the S4 sample, there were larger crystals and less space, which meant that the hydration and hardening process of the calcined gypsum can be accelerated by soluble phosphorus in deep retardation circumstances.

## 4. Conclusions

In this study, the influence of soluble phosphorus on the hydration process and the mechanism of calcined gypsum under deep retarding condition were studied. The data showed that the protein retarder can significantly prolong the setting time of calcined gypsum. The extension of setting time was positively correlated with the dosage and negatively correlated with strength. The co-existence of soluble phosphorus and protein retarders reduced the efficiency of the retarder and decreased the setting time of the system. The strength of the system was also reduced by the soluble phosphorus.

In addition, the hydration process was changed by addition of soluble phosphorus. The induction period was advanced with the extension of acceleration period and deceleration stage by the accumulation and absorption of soluble phosphorus on the surface of the gypsum. HPO_4_^2−^ reacted with Ca^2+^ and decreased the amount of CaSO_4_·2H_2_O. The growth habit of gypsum crystals was changed by soluble phosphorus, while the shape of the crystal was transformed to be coarser with large stumpy and plate-like structures.

## Figures and Tables

**Table 1:** Chemical composition of calcined gypsum (wt./%). LOI is loss on ignition at 85 °C.

CaO	SO_3_	SiO_2_	Al_2_O_3_	MgO	Fe_2_O_3_	Na_2_O	LOI
41.92	52.15	2.72	1.11	0.75	0.51	0.16	6.67

**Table 2:** Fundamental physical properties of the calcined gypsum.

Water Consumption of Normal Consistency (%)	Specific Gravity (g/cm^3^)	Setting Time (min)		Flexural Strength (MPa)		Compressive Strength (Mpa)	
		Initial	Final	2h	1d Dry	2h	1d Dry
56	2.38	4.6	7.4	3.2	7.6	8.6	22.2

**Table 3:** Seven experiment samples.

Sample	Gypsum(wt.%)	W/G	Retarder(wt.%)	P_2_O_5_(wt.%)
S1	100	0.43	0	0
S2	100	0.43	0.02	0
S3	100	0.43	0.04	0
S4	100	0.43	0.06	0
S5	100	0.43	0.06	0.1
S6	100	0.43	0.06	0.3
S7	100	0.43	0.06	0.5

**Table 4:** EDS data.

Dot	Weight of P(%)
1	4.87
2	4.00
3	3.15

## Data Availability

The data presented in this study are available on request from the corresponding author.
